# Supplementary material for: Genetic associations of vitamin D receptor polymorphisms with advanced liver fibrosis and response to pegylated interferon-based therapy in chronic hepatitis C
Source: PeerJ. 2019 Sep 11;7:e7666. doi: 10.7717/peerj.7666 (PMC6744935; doi:10.7717/peerj.7666)
Supplement: Supplemental Information 1 [file peerj-07-7666-s001.docx]

**Supplement table 1: The primer sequences and polymerase chain reaction conditions the studied single nucleotide polymorphisms.**

| **Gene** | **SNP ID** | **primer sequence** | **Temp. annealing (°C)** | **restriction enzyme** | **allele** | **product size (bp)** |
| --- | --- | --- | --- | --- | --- | --- |
| *IL28B* | rs12979860 | F: 5' TAC ACC CGT TCC TGT CCC AAG 3’ | 62 | - | C | 300 |
|  |  | R: 5' CTC TTC CTC CTG CGG GAC AAG 3’ |  |  | T |  |
| *FokI* | rs2228570 | F: 5' TGG CAC TGA CTC TGG CTC TGA 3' | 52 | FokI | C | 266 |
|  |  | R: 5' CTC CCT TCA TGG AAA CAC CTT G 3' |  |  | T | 205, 61 |
| *BsmI* | rs1544410 | F: 5' CTC ACT GCC CTT AGC TCT GC 3' | 58 | BsmI | C | 254, 103 |
|  |  | R: 5' TTG GAC CTC ATC ACC GAC AT 3' |  |  | T | 357 |
| *Tru9I* | rs757343 | F: 5' CTC ACT GCC CTT AGC TCT GC 3' | 58 | Tru9I | G | 357 |
|  |  | R: 5' TTG GAC CTC ATC ACC GAC AT 3' |  |  | A | 264, 94 |
| *ApaI* | rs7975232 | F: 5' TTT GGG GCC AGG CAG TGG TAT 3' | 58 | ApaI | C | 229, 111 |
|  |  | R: 5' CGG TAC TGC TTG GAG TGC TCC TC 3' |  |  | A | 340 |
| *TaqI* | rs731236 | F: 5' TTT GGG GCC AGG CAG TGG TAT 3' | 58 | TaqI | A | 340 |
|  |  | R: 5' CGG TAC TGC TTG GAG TGC TCC TC 3' |  |  | G | 191, 149 |
