# Supplementary material for: Genetic associations of vitamin D receptor polymorphisms with advanced liver fibrosis and response to pegylated interferon-based therapy in chronic hepatitis C
Source: PeerJ. 2019 Sep 11;7:e7666. doi: 10.7717/peerj.7666 (PMC6744935; doi:10.7717/peerj.7666)
Supplement: Supplemental Information 3 [file peerj-07-7666-s003.docx]

| Factor | Code |
| --- | --- |
| Status | 1 Naïve  3 Sustained virological response  4 Non sustained virological response |
| Sex | 1 Male  2 Female |
| HBsAg | 1 Positive  2 Negative  9 Unknown |
| Anti HCV | 1 Positive  2 Negative  9 Unknown |
| PegIFN_3g | 1 PegIFN 2a  2 PegIFN 2b  3 Not treated with PegIFN |
| Peg2a_2g | 1 Yes  2 No |
| Peg2b_2g | 1 Yes  2 No |
| Ribavirin_y | 1 Yes  2 No |
| IL28Brs12979860 | 1 CC  2 CT  3 TT |
| VDR1rs2228570_Fok1 | 1 TT  2 TC  3 CC |
| VDR2rs1544410_Bsm1 | 1 GG  2 GA  3 AA |
| VDR3rs757343_Tru91 | 1 GG  2 GA  3 AA |
| VDR3rs7975232_Apa1 | 1 GG  2 GT  3 TT |
| VDR3rs731236_Taq1 | 1 TT  2 TC  3 CC |
